# Targeting inflammation in heart failure—from genetic evidence to therapeutic agents

**DOI:** 10.1093/eschf/xvaf030

**Published:** 2026-01-08

**Authors:** Mateusz Sokolski, Daniel I Bromage

**Affiliations:** Faculty of Medicine, Institute of Heart Diseases, Wroclaw Medical University, Borowska 213, 50-556 Wroclaw, Poland; Institute of Heart Diseases, University Hospital, Borowska 213, 50-556 Wroclaw, Poland; Cardiac Department, King's College London British Heart Foundation Centre of Excellence, School of Cardiovascular Medicine and Sciences, The James Black Centre, London SE5 9NU, UK; King's College Hospital NHS Foundation Trust, London SE5 9RS, UK

**Keywords:** Inflammation, Heart failure, Mendelian randomization, Inflammatory biomarkers

Inflammation has long been associated with adverse outcomes in heart failure (HF). However, whether inflammation is an important precipitating or exacerbating factor, or simply a conserved protective mechanism in response to myocardial injury, remains unresolved. While several pre-clinical studies support a causal role for immune dysregulation and inflammation, evidence from human studies remains mixed. Nonetheless, inflammation is undoubtedly an important facet of myocardial remodeling, and inflammatory proteins are frequently identified as biomarkers of HF severity.^1,2^ This applies both to dilated cardiomyopathy and ischaemic HF.^3–5^

The association between inflammation and HF severity has also been observed in both HF with reduced ejection fraction (HFrEF) and HF with preserved ejection fraction (HFpEF).^6^ Chronic inflammation, often driven by comorbidities such as obesity and diabetes, strongly contributes to endothelial and microvascular dysfunction, leading to secondary structural and functional cardiac changes typical for HFpEF.^7,8^ An increasing number of analyses confirm these associations, but no direct cause-effect relationship has yet been proven. Moreover, the inflammatory component is recognized in specific types of cardiomyopathies, such as Takotsubo cardiomyopathy, hypertrophic cardiomyopathy, and other genetically determined cardiomyopathies, and it is precisely in this genetic background that we should look for the foothold of inflammatory mechanisms.^9,10^

Zhu *et al.*^11^ evaluate genetically supported causal associations between circulating inflammation-related proteins and HF. Using genome-wide association study (GWAS) summary statistics for 91 proteins from the SCALLOP Consortium (14 824 participants) and HF outcomes from FinnGen (29 218 cases) and HERMES (47 309 cases), they perform a two-sample Mendelian randomization (MR) analysis. In FinnGen, matrix metalloproteinase-1 (MMP-1) and tumor necrosis factor-beta (TNF-β) show positive associations with HF risk, whereas urokinase-type plasminogen activator (uPA) is inversely associated with HF risk in both FinnGen and HERMESl; of these, only the uPA association replicated across datasets. A meta-analysis estimates an overall odds ratio of ∼0.89, consistent with a potential protective effect.

This study strengthens the case for causality by leveraging germline instruments to link specific inflammatory proteins to HF risk, moving the field beyond simple correlation. That said, the focus on a restricted protein panel leaves substantial biology untested, instruments for some targets are sparse and susceptible to residual pleiotropy, and plasma pQTLs may not reflect activity within cardiac or vascular compartments. In addition, heterogeneous HF endpoints limit phenotype-specific conclusions, and the predominantly European ancestry limits generalizability. Even with these caveats, this is a useful study that helps prioritize inflammatory targets for HF and provides data for more phenotype-resolved studies.

The involvement of MMP-1, TNF-β, and uPA is supported by pre-clinical evidence. MMP-1 primarily degrades type I and III collagen, which are key components of the cardiac extracellular matrix.^12^ In a transgenic mouse model, overexpression of human MMP-1 resulted in progressive extracellular matrix degradation, indicating a mechanistic role for MMP-1 in structural cardiac remodeling, and left ventricle diastolic and systolic dysfunction.^13^ TNF-α (predominantly from monocytes/macrophages and T cells) and TNF-β (lymphotoxin-α, mainly from lymphocytes) are pro-inflammatory cytokines. TNF can induce MMP expression, resulting in cardiac collagen degradation and fibrosis. Pharmacological blockade of TNF-α using a soluble TNF receptor led to a significant reduction in atherosclerotic plaque burden, establishing a cause-and-effect relationship between TNF signaling and atherosclerosis.^14^ Similarly, genetic ablation or pharmacological inhibition of uPA (and/or MMP-9) in mouse models of pressure overload attenuated maladaptive ventricular remodeling and preserved cardiac function, highlighting the crucial involvement of the plasminogen activation pathway in adverse myocardial remodeling.^15^ The above results are reflected in clinical and translational studies. Elevated myocardial TNF-α has been reported in ischaemic heart disease, dilated cardiomyopathy, and pulmonary vascular diseases secondary to left-sided HF, and higher concentrations correlate with disease severity.^16^ Elevated MMP-1 has been observed in worsening HF and correlates with pro-inflammatory cytokine expression. Unlike these pathways, uPA showed a potentially beneficial profile. It is a serine protease and plasminogen activator that promotes cell trafficking, facilitating leukocyte extravasation and monocyte/macrophage migration, and supports matrix remodeling during tissue repair.^17^ These pleiotropic roles suggest a complex contribution to cardiovascular disease, with evidence for involvement in atherosclerotic plaque formation and destabilization. Furthermore, in experimental viral myocarditis, inhibition of uPA or MMP activity attenuates cardiac injury. In contrast, in the present study, higher uPA levels were associated with a lower risk of HF. The mechanisms linking uPA to HF risk remain unclear. As a plasminogen activator, uPA contributes to fibrinolysis and may modulate thrombo-inflammatory pathways, but definitive cardioprotective effects in HF have not been established. Interpretation should also consider the balance between uPA and its inhibitor PAI-1, and differences between circulating and tissue activity. Its impact is likely to be context-dependent, according to HF aetiology and concomitant risk factors such as disordered lipid metabolism, underscoring the need for phenotype-specific analyses.

Whether these findings can translate into clinical practice is an outstanding, but crucial, question. Early anti-TNF trials were disappointing. For example, in ATTACH, infliximab transiently lowered CRP and IL-6 in moderate–severe HF but did not improve outcomes, and higher dosing was associated with harm.^18^ Non-selective MMP inhibition, most commonly with doxycycline, has been explored in small post-myocardial infarction (MI) and peri-operative studies, showing modulation of MMP activity and remodeling signals but no definitive HF benefit,^19^ and there are no phase III studies. Selective MMP inhibitors remain in the early stages of development. Targeting the uPA/uPAR axis is being developed in oncology but, given mixed cardiovascular data, has not been pursued in HF.

Despite these disappointments, there are data suggesting that treating inflammation in HF may be fruitful. Canakinumab, an IL-1β inhibitor, reduced recurrent events, including HF admissions, and inflammatory biomarkers in patients with prior MI,^20^ while colchicine early post-MI has shown mixed but encouraging results.^21^ As is known, IL-6 may play an important role in the development of HF, and IL-6 blockade may provide anti-inflammatory, antifibrotic, and partially antioxidative effects.^7,8^ Building on targeting IL-6, the ongoing ARTEMIS trial (NCT06118281) is now testing whether targeting IL-6 signalling with ziltivekimab can reduce post-MI HF, and the HERMES trial (NCT05636176) is testing it in HF with preserved or mildly reduced ejection fraction.

The genetic signals in the present study do not map neatly onto pathways with the strongest translational traction. MR captures lifelong variance in circulating proteins, not disease-, tissue- or proteoform-specific activity that drives acute injury and chronic remodeling. Added to this, heterogeneous HF endpoints blur distinct phenotypes and therapeutic windows; whereas positive trials have typically emerged when time-windows are narrow (early post-MI) and inflammation-enriched phenotypes are targeted. The remedy may be deep phenotyping of selected patients at defined timepoints, using serial proteomics and cytokines, cell-resolved immune profiling and imaging, embedded within intervention studies.

The results of this study, and others in this field, show that inflammatory biomarkers can stratify risk in HF. MR analyses such as that by Zhu *et al.*^11^ extend this by suggesting a causal contribution of inflammation to HF. Bridging this to intervention will, however, require deep phenotyping of selected patients at defined timepoints to nominate actionable, phenotype-specific targets.

## Declarations

### Data Availability

No data were generated or analysed for this manuscript.
